# Nuclear matrix associated RNAs in posterior silk glands show developmental dynamics in *Bombyx mori* in 5th instar larvae

**DOI:** 10.1186/s13104-022-05951-2

**Published:** 2022-02-19

**Authors:** Alekhya Rani Chunduri, Anugata Lima, Resma Rajan, Anitha Mamillapalli

**Affiliations:** Department of Biotechnology, Institute of Science, GITAM (Deemed To Be University), Visakhapatnam, Andhra Pradesh 530 045 India

**Keywords:** Nuclear matrix, RNA, *Bombyx mori*, Posterior silk glands, Repeats, Chromatin

## Abstract

**Objectives:**

The nuclear matrix maintains and regulates chromatin structure. RNA is an integral component of the nuclear matrix and is essential to its structural maintenance. *Bombyx mori* is a major economic contributor in the sericulture industry and produces fibroin—the most important silk protein in its posterior silk glands during 5th instar larval stage. The present study investigates the composition of nuclear matrix RNA prepared from the posterior silk glands of *Bombyx mori* during fifth instar larval stage where maximum silk production occurs. The datasets from which the analysis is carried out are part of data note titled “Nuclear matrix associated RNA datasets of posterior silk glands of *Bombyx mori* during 5th instar larval development”.

**Results:**

The results showed significant enrichment of nuclear matrix RNA from day 1, to day 5 and day 7. Nuclear RNA showed increased abundance from day 1 to day 5 and day 7. Nuclear matrix RNA exhibited repetitive RNA sequences, of which UGUCC and GCUGGU were the most abundant. Genes involved in metabolic pathways showed significant enrichment correlating with silk production. These results emphasize the role of dynamic, repetitive DNA transcripts in chromatin architecture and further reveal the close association between the nuclear matrix and gene expression.

**Supplementary Information:**

The online version contains supplementary material available at 10.1186/s13104-022-05951-2.

## Introduction

The nuclear matrix (NuMat) is the non-chromatin residual nuclear structure, which remains following nuclease treatment and salt extraction of isolated nuclei. Although, it consists of DNA, RNA, and protein, the RNA component is considered its major constituent [1]. In *Drosophila melanogaster* embryos, the NuMat extract retained 30% of RNA compared to DNA (1–2%) and protein (10%) [2]. In this study, we investigated nuclear retention of RNA obtained from NuMat preparations of posterior silk glands (PSGs) from 5th instar *Bombyx mori* larvae, on days 1, 5, and 7 of development.

Nuclear matrix associated RNA (NuMat RNA) contributes to gene regulation and genome stability [3]. The non-coding RNA associated or transcribed from repeat sequences were found to play an essential role in heterochromatin formation [4]. Many transcription sites are associated with distal or inter-chromosomal chromatin-associated RNAs [5]. These findings highlight the importance of NuMat RNA in regulating gene expression. Many repeat sequences have been associated with the nuclear matrix [6, 7] and have shown to contribute to the stability and viability of the nuclear matrix [6].

Simple sequence repeats (SSR) of long non-coding RNA (lncRNA) recruit pyrimidine tract binding protein-specific motifs in the perinucleolar compartment, thereby regulating pre mRNA splicing and cell survival [8]. lncRNAs in particular, play an important role in aiding or hindering interactions of macromolecules by acting as scaffolds or decoys. They regulate nuclear processes such as chromosome topology, chromatin state and gene transcription [9].

The silkworm, *B. mori* is an economically significant insect that largely contributes to silk production. The PSGs are the sites of synthesis for fibroin, the most important protein involved in silk production. Here, we determined the composition and abundance of genome-wide NuMat RNAs in the PSGs of 5^th^ instar *Bombyx mori* larvae, on days 1, 5, and 7. In this study, we identified microsatellite repeats/simple sequence repeats in all 3 days. In addition, we identified genes associated with nuclear matrix RNAs and their related biological pathways. Finally, we determined the functional role of each of the genes.

## Materials and methods

### Isolation and quantification of nuclear matrix RNA

Fresh mulberry leaves (V1 variety) were used to feed 100 *B. mori* larvae of CSR2X CSR4 variety throughout the 5th instar stage. PSGs were dissected from 5th instar larvae on day 1, day 5, and day 7 under sterile conditions using 1X PBS. PSGs were pooled [day1 (n = 30), day 5 (n = 15) and day 7 (n = 10)] from a single rearing and were homogenized in nuclear isolation buffer (5 times volume of the weight of the tissue) and processed for nuclei and nuclear matrix isolation by following the standard protocol for isolation through nuclease digestion and salt extraction [2].

The nuclear and nuclear matrix pellets were then used for RNA isolation with TRIzol reagent [10]. Quantification of nuclear and nuclear matrix PSG RNA on day 1, day 5, and day 7 was carried out by UV spectrophotometry. Student’s t-test: two-sample assuming unequal variances, was used to determine significance of variation. Agarose gel electrophoresis was performed to determine the NuMat RNA.

### Matrix RNA-sequence analysis

PSG NuMat RNA datasets of 5th instar day 1 (SG 1), day 5 (SG 5) and day 7 (SG 7) were acquired from NCBI SRA database [11–13]**.** These datasets were mapped data against *B. mori* reference genome (Bombyx mori (assembly Bmori_2016v1.0), https://www.ncbi.nlm.nih.gov/genome/?term=txid7091[Organism:noexp]) using the ‘BOWTIE2’ tool (Additional file 3: Table S1). SSR prediction was carried out with the mapped datasets (SG 1, SG 5 and SG 7) using the MISA software. Gene identification and quantification was carried out based on mapped data. The total aligned reads to genes were counted using HTSeq. The read counts for individual genes were used to construct the expression profile at gene level. A total of 9623, 8042 and 8499 expressed genes were found in the three datasets. The “diamond” tool (https://github.com/bbuchfink/diamond) was used to perform BLAST of the input nucleotide sequences (gene sequences of all the expressed genes fetched from *B. mori* genome [ https://www.ncbi.nlm.nih.gov/genome/?term=txid7091[Organism:noexp]) using blastx against the Insecta protein sequences downloaded from Uniprot database (subject sequences) (https://www.uniprot.org/uniprot/?query=taxonomy:50557).

The GOs were mapped against expressed transcripts. KAAS (KEGG Automatic Annotation Server) was used and BLASTx (with parameters: e-value 1e-05 and minimum similarity > 30%) was performed for query sequences (amino-acid sequences of the protein-coding genes) which were aligned against the *B. mori* reference genome (Bombyx mori (assembly Bmori_2016v1.0), https://www.ncbi.nlm.nih.gov/genome/?term=txid7091[Organism:noexp]) to identify the biological pathways associated with the expressed genes. The biological functions associated with these genes were also determined.

## Results and discussion

**Fig. 1:**
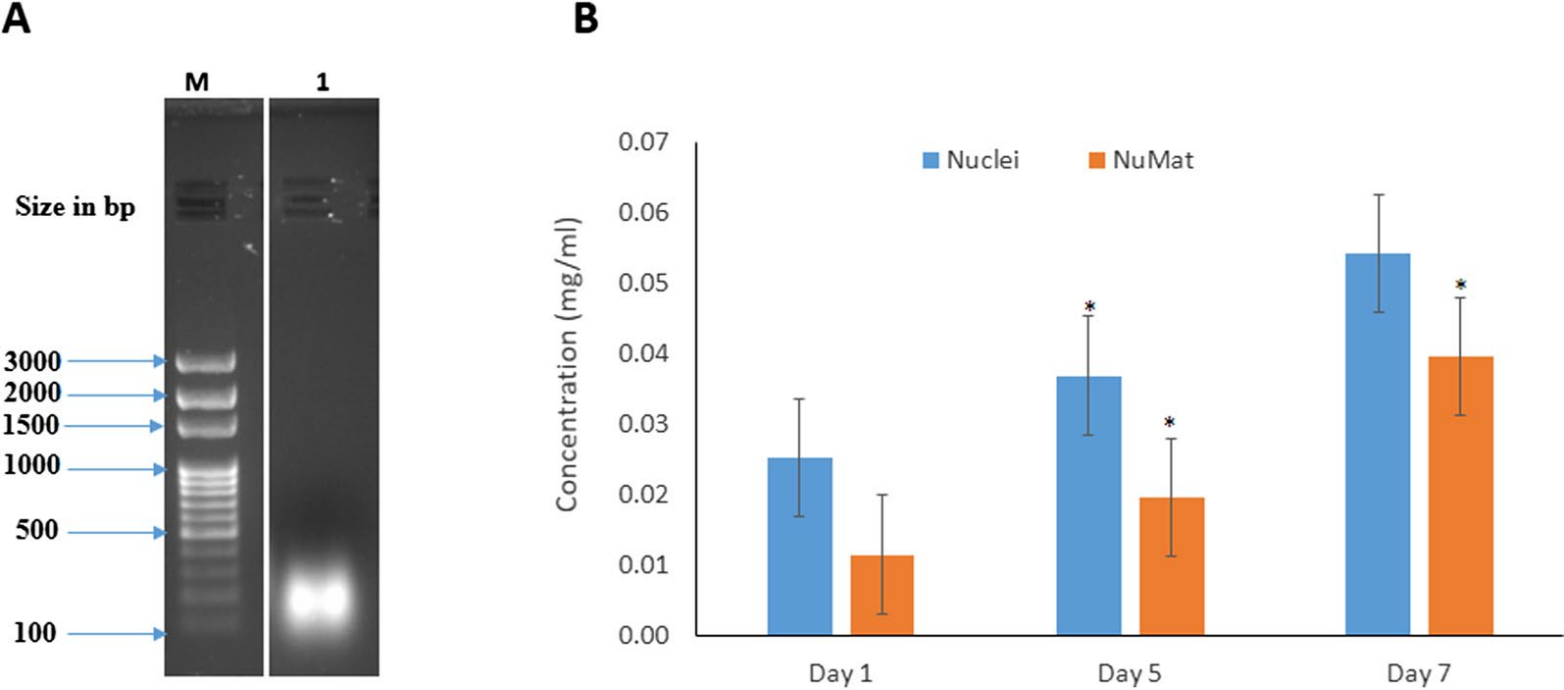
Isolation of nuclear matrix RNA from 5th instar posterior silk glands of *B. mori.* **A** A representative image of the day 5 NuMat RNA (1) separated on 1.2% agarose gel alongside a 100 bp DNA ladder (M). The marker lane and the day 5 NuMat RNA were selected from the full image given in Additional file 1: Fig. S1. **B** The nuclear and NuMat RNA of day 1, day 5, and day 7 were quantified using UV spectrophotometry (day 1: n = 30, day 5: n = 15, day 7: n = 10). Student’s t-test assuming unequal variances was used for statistical analysis. Asterisk shows significant variation (*p* < 0.05). The nuclear retention of RNA in the nuclear matrix was identified by comparing the nuclear and NuMat RNA concentrations on different days of 5th instar development. The nuclear RNA increased significantly from day 1 to day 5 (as represented by the asterix on the blue bar of day 5 on the graph). The NuMat RNA increased significantly from day 1 to day 5 and from day 5 to day 7 (as represented by the asterix on the orange bars of day 5 and day 7 in the graph)

The genomic organisation, its regulation and the processes in regard to its functioning are part of the nucleus [14–17]. The placement of elements responsible for genetic regulation is specific to the relevant developmental stage. Nuclear matrix RNA, a major constituent of the nuclear framework regulates gene expression during development via organisational modifications and structural composition [18–22]. Gene expression is linked to the spatial and temporal organisation of the genes facilitated by their anchoring to the nuclear matrix. RNA maintains chromatin structure and regulates gene expression [23, 24]. A study on the developmental stage-specific expression of fibroin in PSGs of *B. mori*, found considerable increase in the expression levels of fibroin RNA during the 5th instar compared to the 4th instar larval stage [25]. However, the NuMat RNA levels in the PSG of *B. mori* have not been studied. To measure the abundance and size of RNA associated with the nuclear matrix of *B. mori*, nuclear and NuMat RNA were isolated from PSGs at day 1, day 5, and day 7 of development. Agarose gel electrophoresis was performed without formamide and formaldehyde to check the RNA enrichment. The data showed that NuMat RNA approximately ranged in size from 100 to 400 bp (Fig. 1A; Additional file 1: Fig. S1). While the agarose gel electrophoresis results show the enrichment of RNA, the precise determination of the size of NuMat RNA requires a more sensitive approach. Therefore, TapeStation analysis of the NuMat RNA libraries of the three datasets was carried out prior to sequencing which showed the size to be between 200 to 1000 bp (Additional file 2: Fig. S2). The amount of nuclear RNA showed a significant increase from day 1 to day 5 (*p* < 0.05) but no significant increase from day 5 to day 7 (p > 0.05). This correlates with the increase in protein expression observed on day 5 of the 5th instar larval development. The amount of nuclear RNA retained in the nuclear matrix on day 1, 5, and 7 were 33.33%, 50% and, 80% respectively. The NuMat RNA showed a significant increase in concentration from day 1 to day 5 (*p* < 0.05) and from day 5 to day 7 (*p* < 0.05) (Fig. 1B). It is interesting to note that an increase in concentration of NuMat RNA on day 7 (towards the wandering stage) was observed despite no significant change in concentration of nuclear RNA from day 5 to day 7. This is likely because the role of NuMat RNA in regulation of gene expression during larval to pupal transition on day 7 marks the end of the 5th instar. The influence of NuMat RNA on fibroin gene expression may also be a factor, as maximum silk is produced during the wandering stage to prepare for cocoon formation.

**Fig. 2:**
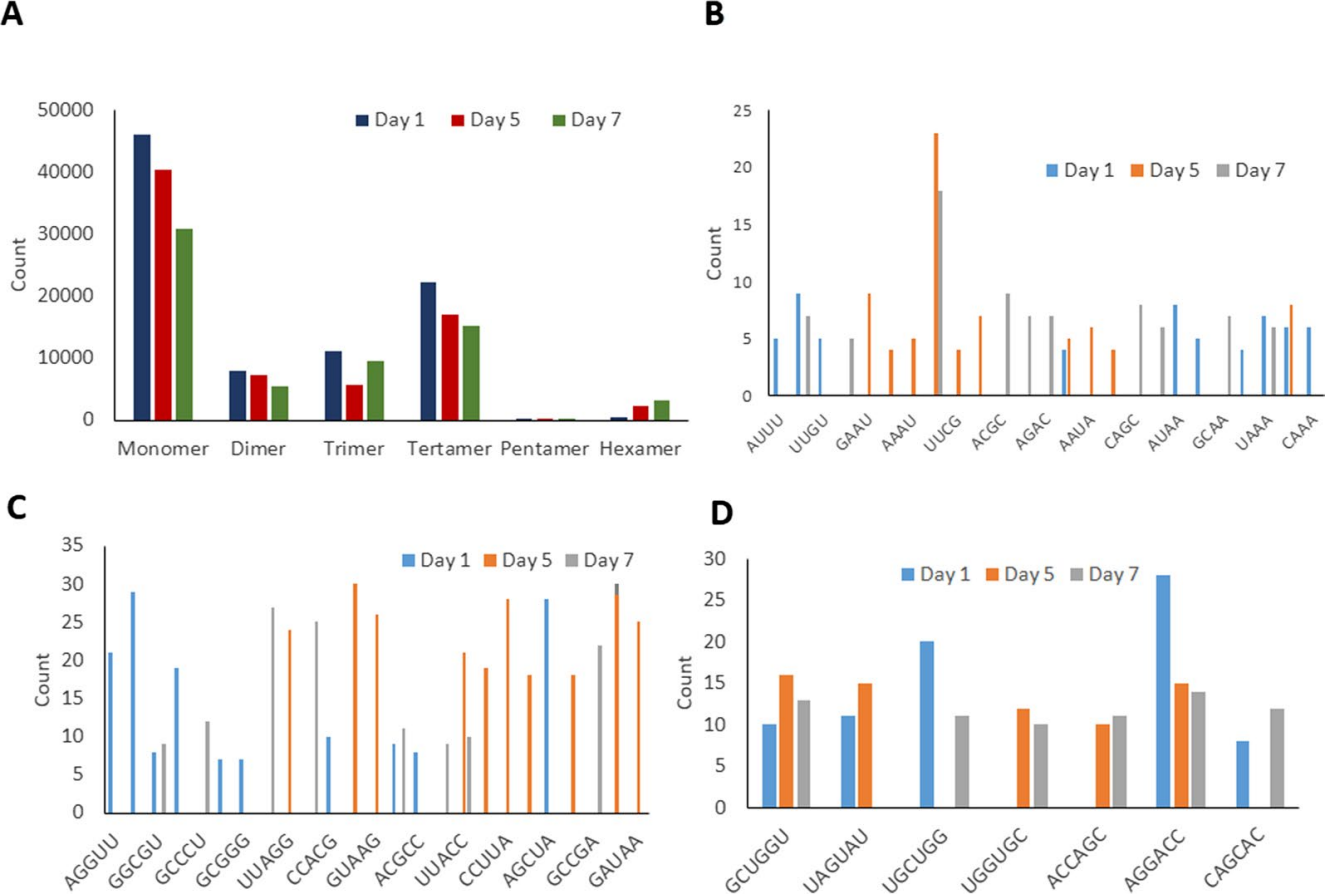
SSR Prediction. **A** Comparison of the types of SSRs identified. **B** Tetra-nucleotide (**C**) Penta-nucleotide and (**D**) Hexa-nucleotide repeats identified in the SG 1, SG 5, and SG 7 nuclear matrix RNA datasets

**Fig. 3:**
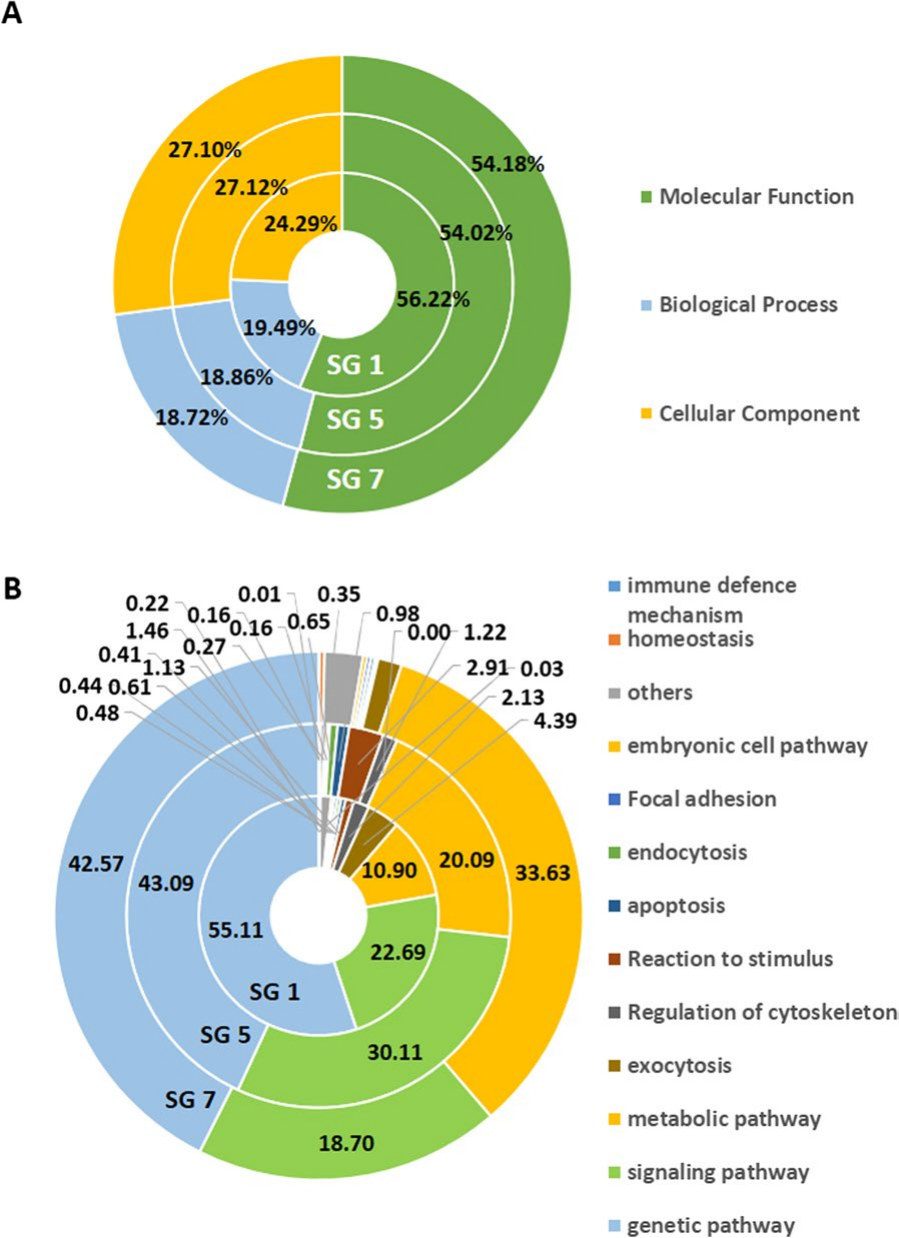
**A** Gene ontology and biological pathways. The functions of the expressed genes are shown in day 1, day 5 and day 7 nuclear matrix RNA of 5th instar *B. mori* PSGs. **B** The pathways associated with those of the identified expressed genes in the three datasets (SG 1, SG 5, and SG 7) are shown

To study the composition of NuMat RNA repeats, SSRs were determined for all 3 days (Additional file 4: Table S2). SSRs are short repeating chains that include a repeating unit of 1–6 bp, and are essential for maintenance and vitality of NuMat structure and gene expression. In *D. melanogaster*, a novel lncRNA with AAGAG repeats in NuMat was found to be essential in maintaining structural organisation of interphase chromosomes and compartmentalising of nuclear organelle. It was also shown to be indispensable for pupal formation and survival [6]. In this study, the mono-nucleotides, tri-nucleotides and tetra-nucleotides were most abundant during 5th instar development while the penta-nucleotide repeats were fewer in comparison (Fig. 2A). On day 1, the repeats CUUU, UUUU, UUGGU, UGCUU, UGCUCC and GCUGGU were most abundance. On day 5, the repeats CUGG, the telomeric repeat CCUUU and the repeats UUUUG and GCUGGU were most abundant, and on day 7, UCGC, CUGG, UCGG, GCCGU, UGCUCC were the most abundant (Fig. 2B–D). It is noteworthy that the TTAGG/CCUAA transcript of telomeric repeat found on day 5 is conserved in insects and is regularly interrupted by non-LTR retrotransposon elements in *B. mori* [26]*.* The repeats UGCUCC and GCUGGU occurred abundantly on all three days, suggesting a role for general structural or functional maintenance in the NuMat. The change in the composition and abundance of nuclear matrix-associated repeats from day 1 to day 5 and day 5 to day 7 highlight the dynamic nature of the nuclear matrix structure. SSRs were found to be highly enriched in all three datasets and a high variation in the SSRs was observed in all three days of the 5th instar in accordance with the high complexity and multi-factorial regulation of the posterior silk glands. Furthermore, the genes associated with the NuMat RNA, were identified from the three developmental datasets SG 1, SG 5, and SG 7. GO analysis revealed that half of the genes (56.22%, 54.02% and 54.18% in day 1, day 5 and day 7, respectively) had molecular functions. These included, metal ion binding—particularly magnesium, calcium and zinc ion binding, nucleic acid binding—specifically DNA binding functions, nucleotide binding, helicase activity, RNA–DNA hybrid ribonuclease activity, RNA directed DNA polymerase activity, ATP binding, sequence-specific DNA binding transcription factor activity, transcription cofactor, coactivator and co-repressor activity, RNA binding, structural constituent of ribosome, translation initiation factor activity, translation elongation and release factor activities, catalytic activity, GTPase activity, oxidoreductase activity, transferase and phosphotransferase activity, hydrolase activity, and transmembrane transporter activity. These functions imply that the NuMat RNA is likely intricately involved in transcription. There is an increase in the number of NuMat RNA linked genes associated with cellular components from day 1 to day 5 that are maintained at day 7 (Fig. 3A). Further, downstream analysis associated with the expression data revealed that most of the genes were involved in metabolic, signaling and genetic pathways (Fig. 3B). The most abundantly occurring pathways are the genetic pathways (55.11%, 43.09% and 42.57% in day 1, day 5, and day 7, respectively) which decreased from day 1 to day 7. The genetic pathways associated with the NuMat included chromosomal and associated proteins, basal transcription factors, mRNA biogenesis, transcription machinery, RNA polymerase and translation factors; further validating the role of NuMat RNA in the regulation of transcription and translation. The signaling pathways increased from day 1 to day 5 but significantly decreased from day 5 to day 7. The metabolic pathways however see a steady and clear increase from day 1 to day 5 to day 7. Genes involved in apoptosis, endocytosis, lysosome, dorso-ventral axis formation, DNA replication, etc., were also found in abundance which correlate with larval to pupal transition at the end of 5th instar and the beginning of cocoon formation. Similar results by earlier studies showed an increase in the expression of genes associated with apoptosis and autophagy for preparation of metamorphosis. The analysis of apoptosis related genes during larval to pupal transition showed an increased transcription of BmDredd in MSGs and PSGs of *B. mori* participating in silk gland degradation [27]. Similarly, the expression BmEcR, found upstream of BmDredd, along with BmE74A and Bm Br-c was responsible for triggering autophagy and apoptosis pathways in the silk glands [28]. These studies correlate with the increased expression of apoptopic genes supporting pupal transition found in our study.

Our results show that the nuclear matrix RNA is highly repetitive and dynamic in nature underscoring its role in chromatin architecture and gene expression.

## Limitations

The data used for analysis in this paper was obtained from a single rearing by pooling PSGs of CSR2XCSR4 strain of *Bombyx mori*.

## Supplementary Information

**Additional file 1: Fig. S1.** Agarose gel electrophoresis. The uncropped image for the day 5 NuMat RNA (1) separated on 1.2% agarose gel alongside a 100 bp DNA ladder (M) is provided.

**Additional file 2: Fig. S2. **TapeStation profiling of NuMat RNA. (A) TapeStation Ladder, (B) day 1, (C) day 5 and (D) day 7 NuMat RNA of PSG NuMat RNA during 5^th^ instar development. The NuMat RNA is analysed by TapeStation profiling and the size range is shown.

**Additional file 3: Table S1.** Alignment statistics of SG 1, SG 5, and SG 7 datasets. The percentage of reads mapped against the reference genome is shown.

**Additional file 4: Table S2. **Prediction of SSRs in SG 1, SG 5 and SG 7 PSG datasets. The simple sequence repeats are identified using MISA software from the three nuclear matrix RNA datasets.

## Data Availability

The data described in this research note can be freely and openly accessed on National Centre for Biotechnology Information (NCBI), in the Sequence Read Archive under the accessions and identifiers: SRX9472457 (https://identifiers.org/ncbi/insdc.sra:SRX9472457) for day 1, SRX7730613 (https://identifiers.org/ncbi/insdc.sra:SRX7730613) for day 5 and SRX9495290 (https://identifiers.org/ncbi/insdc.sra:SRX9495290) for day 7 data sets. Please see references [11–13] for details and links to the data.

## Abbreviations

NuMat: Nuclear matrix
SSRs: Simple sequence repeats
lncRNA: Long non-coding RNA
PSGs: Posterior silk glands
NuMatRNA: Nuclear matrix associated RNA
GO: Gene ontology
KAAS: KEGG Automatic annotation server
SG1: PSG NuMat RNA dataset day 1
SG5: PSG NuMat RNA dataset day 5
SG7: PSG NuMat RNA dataset day 7
