# Supplementary figures and images for: Nuclear matrix associated RNAs in posterior silk glands show developmental dynamics in Bombyx mori in 5th instar larvae
Source: BMC Res Notes. 2022 Feb 19;15:68. doi: 10.1186/s13104-022-05951-2 (PMC8858543; doi:10.1186/s13104-022-05951-2)

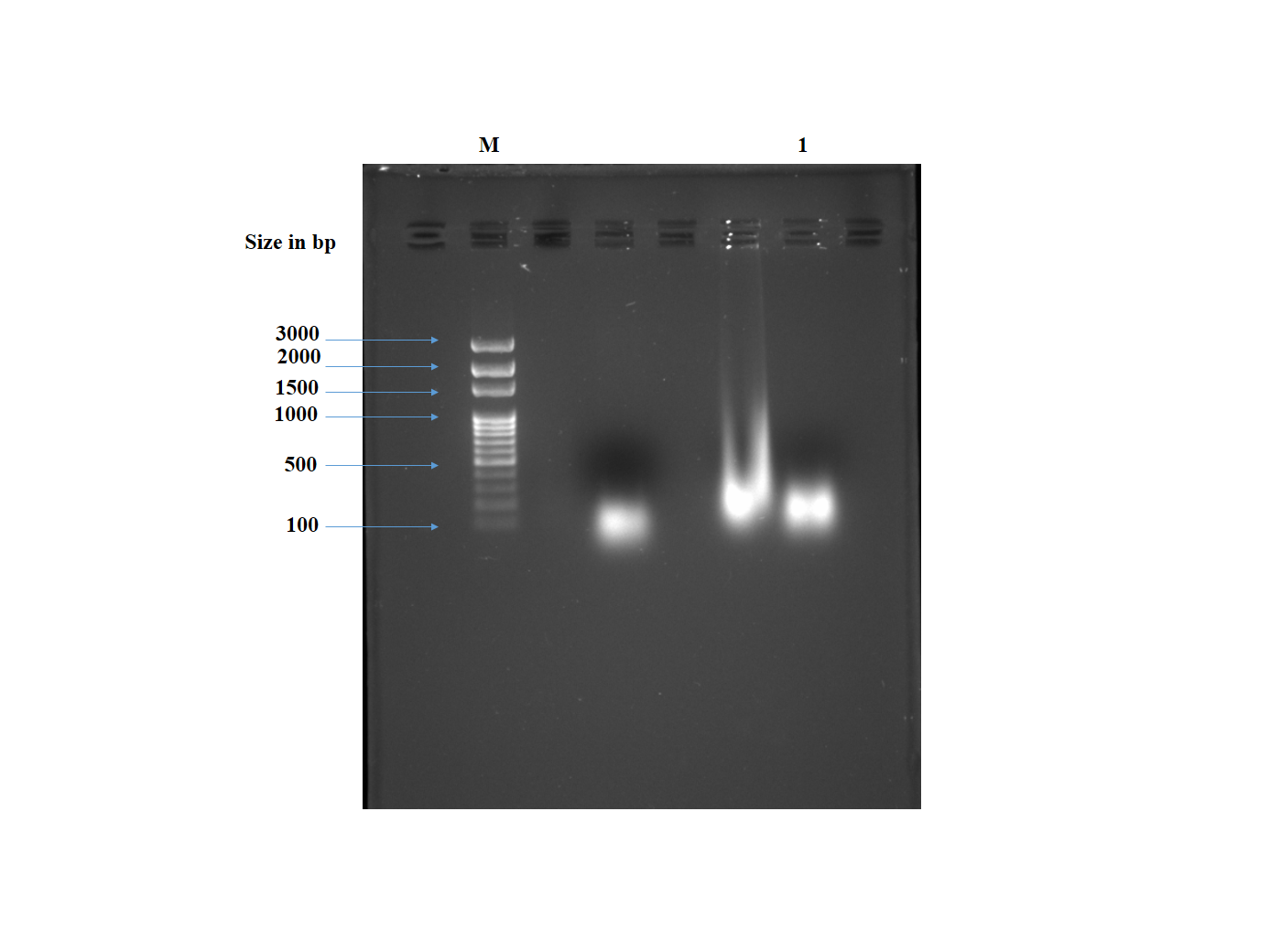

Supplement: Supplementary file 1 — Additional file 1: Fig. S1. Agarose gel electrophoresis. The uncropped image for the day 5 NuMat RNA (1) separated on 1.2% agarose gel alongside a 100 bp DNA ladder (M) is provided. [file 13104_2022_5951_MOESM1_ESM.tif]

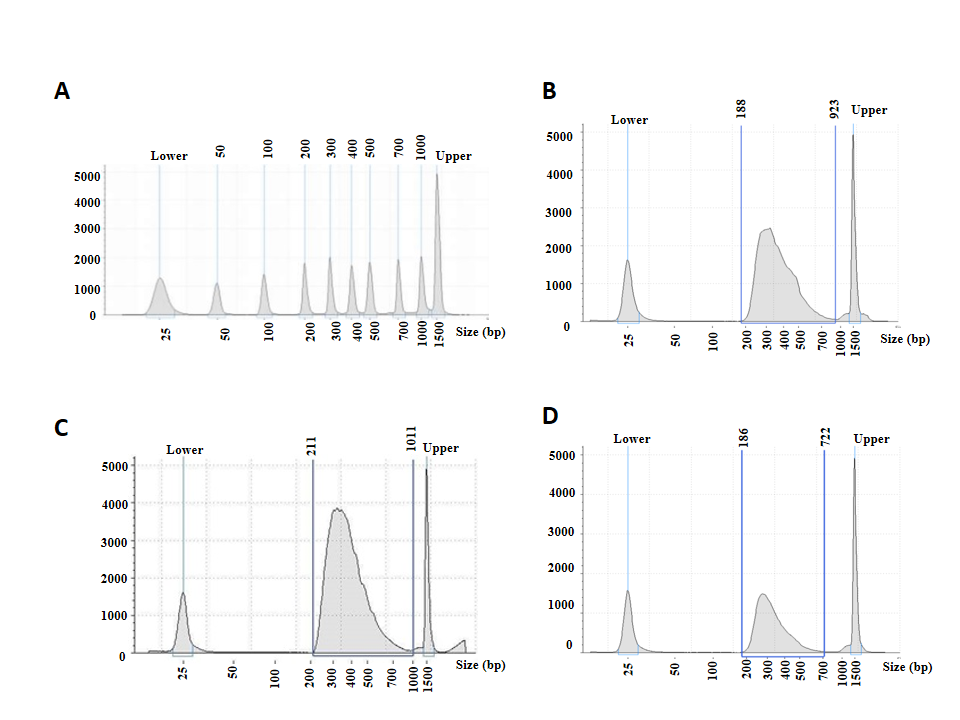

Supplement: Supplementary file 2 — Additional file 2: Fig. S2. TapeStation profiling of NuMat RNA. (A) TapeStation Ladder, (B) day 1, (C) day 5 and (D) day 7 NuMat RNA of PSG NuMat RNA during 5th instar development. The NuMat RNA is analysed by TapeStation profiling and the size range is shown. [file 13104_2022_5951_MOESM2_ESM.tif]
