# Supplementary material for: Nuclear matrix associated RNAs in posterior silk glands show developmental dynamics in Bombyx mori in 5th instar larvae
Source: BMC Res Notes. 2022 Feb 19;15:68. doi: 10.1186/s13104-022-05951-2 (PMC8858543; doi:10.1186/s13104-022-05951-2)
Supplement: Supplementary file 3 — Additional file 3: Table S1. Alignment statistics of SG 1, SG 5, and SG 7 datasets. The percentage of reads mapped against the reference genome is shown. [file 13104_2022_5951_MOESM3_ESM.docx]

**Additional Table 1.**

| **Samples** | **% of reads aligned against reference genome** |
| --- | --- |
| **Day 1** | **91.24** |
| **Day 5** | **87.38** |
| **Day 7** | **87.03** |
