# Supplementary material for: Nuclear matrix associated RNAs in posterior silk glands show developmental dynamics in Bombyx mori in 5th instar larvae
Source: BMC Res Notes. 2022 Feb 19;15:68. doi: 10.1186/s13104-022-05951-2 (PMC8858543; doi:10.1186/s13104-022-05951-2)
Supplement: Supplementary file 4 — Additional file 4: Table S2. Prediction of SSRs in SG 1, SG 5 and SG 7 PSG datasets. The simple sequence repeats are identified using MISA software from the three nuclear matrix RNA datasets. [file 13104_2022_5951_MOESM4_ESM.docx]

**Additional Table 2.**

| **Statistic** | **Day 1** | **Day 5** | **Day 7** |
| --- | --- | --- | --- |
| Number of SSR containing sequences | 82551 | 65256 | 59505 |
| Number of sequences containing more than 1 SSR | 4525 | 6097 | 3757 |
| Number of compound SSRs(i.e., c) | 5245 | 7443 | 4748 |
| Mononucleotides | 46044 | 40400 | 30787 |
| Dinucleotides | 7921 | 7270 | 5510 |
| Trinucleotides | 11038 | 5793 | 9532 |
| Tetranucleotides | 22218 | 16981 | 15154 |
| Pentanucleotides | 116 | 82 | 110 |
| Hexanucleotides | 506 | 2222 | 3181 |
